# Prevalence and Predictors of Overweight and Obesity Among Adolescents in Seremban, Negeri Sembilan, Malaysia

**DOI:** 10.7759/cureus.21795

**Published:** 2022-01-31

**Authors:** Wai Kent Lai, Sherina Mohd Sidik, Rampal Lekhraj, Wan Ying Gan, Siti Irma Fadhilah Ismail

**Affiliations:** 1 Centre for Nutrition Epidemiology Research, Institute for Public Health, Shah Alam, MYS; 2 Department of Psychiatry, Universiti Putra Malaysia, Serdang, MYS; 3 Department of Community Health, Universiti Putra Malaysia, Serdang, MYS; 4 Department of Nutrition, Universiti Putra Malaysia, Serdang, MYS

**Keywords:** associated factor, predictor, prevalence, adolescent, obesity, overweight

## Abstract

Introduction: Obesity is recognized as a serious public health threat. Recent evidence has warned of the alarming rise in the prevalence of childhood overweight and obesity throughout the world. This study aimed to determine the prevalence of overweight and obesity, and its associations with socio-demographic, behavioral, and psychosocial factors among school-going adolescents in Seremban, Negeri Sembilan.

Methods: Cross-sectional study was conducted. A total of 2,221 adolescents were randomly selected from eight secondary schools. A questionnaire was administered to assess socio-demographic profiles, meal patterns, physical activity level, self-efficacy, self-esteem, body size satisfaction, perception of body weight status, depression, anxiety, stress, and nutrition knowledge. Bodyweight and height were measured and BMI-for-age z scores were computed to determine the body weight status. Bivariate analysis and multivariate logistic regression were used for the data analysis.

Results: The prevalence of overweight among the participants in this study was 17.0%, while the prevalence of obesity was 14.9%. The significant predictors of overweight and obesity in this study were breakfast skipping, low physical activity level, low self-efficacy scores in terms of healthy eating, weight and physical activity, body dissatisfaction, and perception of large body size.

Conclusion: The results emphasize the need to broaden the scope of nutrition guidelines, public health policies, and programs to address overweight and obesity among adolescents in Malaysia. The findings also suggest that health education programs should cover practical advice for modifying healthy eating behaviors, increasing physical activity, as well as matters on body image and body satisfaction.

## Introduction

The prevalence of obesity and age-standardized mean body mass index (BMI) increased globally in children and adolescents from 1975 to 2016, particularly accelerated in east, south, and south-east Asia countries [1]. Obesity is commonly recognized as a consequence of a disparity between energy consumption and expenditure, with a rise in positive energy balance being closely associated with the lifestyle adopted and dietary intake preferences [2]. Obesity is associated with a wide range of adverse health consequences, including problems and disorders related to sleeping, respiratory, gastrointestinal, endocrine, nervous system, orthopedic and psychiatric, as well as cardiovascular risk factors [3]. Childhood overweight and obesity often persist into adulthood and thus increase the risks of developing non-communicable diseases at an earlier age [4]. Besides, childhood overweight or obesity is associated with psychological comorbidities, such as depression, lower perceived health-related quality of life, emotional and behavioral disorders, and low self-esteem [5].

In Malaysia, the main factors of nutrition transition are associated with the food environment, lifestyle and behavior changes as well as government policies [6]. Findings from the National Health and Morbidity Survey (NHMS) in 2019 revealed that the prevalence of childhood obesity has increased substantially to 14.8% [7]. There is a lack of the prevalence of overweight and obesity and its association with socio-demographic, behavioral, and psychosocial factors. Therefore, this study aimed to explore the prevalence of overweight and obesity, and the possible associations and contributions of socio-demographic, behavioral, and psychosocial factors in Malaysian school-going adolescents.

## Materials and methods

Study design and participants

A cross-sectional study was conducted in government secondary schools in Seremban, Negeri Sembilan, between February and June 2019. Two-stage cluster sampling was used. In stage one, eight out of 46 secondary schools were randomly selected by using the systematic random sampling method. In stage two, classes were randomly selected by using simple random sampling method to reach the sample size allocated for each form in those selected schools. Secondary school students aged 12 to 18 years (form one, two and four) in the participating schools were eligible. Form three and five students were in their public examination years, therefore they were not included in this study. The exclusion criteria for this study were students attending special education classes for the disabled and students with mental or physical disabilities. The sample size for this study was calculated by comparing the proportion of the prevalence of obesity among males and females (18.9% and 12.3%, respectively [8]), α = 0.05 and 95% power of study. The required sample size was 948. The final sample size was 2,370 after considering 20% of non-response rate and design effect. Self-administered questionnaires were distributed to the students and assistance was given if necessary by the researchers. The total estimated time to complete the questionnaire was about 45 to 55 minutes.

Socio-demographic profile

Information regarding socio-demographic characteristics including date of birth (age), gender, ethnicity and grade were obtained.

Meal pattern

The meal patterns among school students were assessed by the questionnaire, which was adapted from the Adolescent Nutrition Survey 2017 questioning the frequency of consuming breakfast, fast food, outside food, and snacking [9].

Physical activity and screen time

The Physical Activity Questionnaire for Older Children (PAQ-C) was used to evaluate the physical activity level throughout the previous seven days at various times and locations, such as at school, after school and on weekends [10]. PAQ-C contained 10 questions. The first question asked about the participants’ involvement in spare time activities over the previous seven days. For the second to eighth items, participants were questioned about their participation in physical activity during different occasions, for instance, physical education (PE) class, lunch, right after school, evening, weekends, and leisure time over the last week. For the ninth item, participants were questioned about their involvement in daily physical activity over the previous seven days. For the tenth item, participants with uncommon physical activity due to illness or barriers in doing regular physical activities during the past seven days were identified. The PAQ-C summary activity scores were computed by calculating a mean score of all nine items. Higher scores indicated greater levels of physical activity and vice versa. Participants were categorized into three groups based on their PAQ-C summary activity scores: “low physical activity” (with a score ranging from 1.00 to 2.33), “moderate physical activity” (with a score ranging from 2.34 to 3.66), and “high physical activity” (with a score ranging from 3.67 to 5.00).

Screen time was assessed for the duration of television viewing, playing video games, using a computer or accessing the internet during weekend and schooling days. The classification of the duration was based on the Adolescent Nutrition Survey 2017 [9].

Healthy eating and weight self-efficacy

The Healthy Eating and Weight Self-Efficacy scale (HEWSE) was used to evaluate the self-efficacy in terms of healthy eating and weight in which people's confidence in their capability to cultivate and sustain a healthy eating and weight pattern [11]. This questionnaire consisted of 11 questions that were related to two factors, namely healthy food consumption and healthy weight. Individuals answering the questions of the HEWSE scale were requested to rate their confidence in their ability to deal with healthy eating behaviors using a Likert scale ranging from one (strongly disagree) to five (strongly agree). Higher total scores indicated greater levels of self-efficacy. This scale had good internal consistency for the overall (α = 0.82), factor of healthy food consumption factor (α = 0.81), and factor of healthy weight (α = 0.82).

Perceived self-efficacy scale for physical activity

Perceived Physical Activity Self-Efficacy Scale for Adolescents was used to assess self-efficacy for physical activity [12]. Participants were required to rate their confidence in their ability to overcome specific barriers to physical activity. There were 11 items on this scale and the rating was based on a four-point scale from one (not true at all) to four (very true). A higher score indicated a greater level of self-efficacy in overcoming barriers. Cronbach’s alpha coefficient for the overall self-efficacy measure was 0.86.

Self-esteem

Participants’ self-esteem was assessed using a 10-item Rosenberg Self-Esteem Scale [13]. All items were answered using a 4-point Likert scale ranging from three (strongly agree) to zero (strongly disagree). Items 3, 5, 8, 9 and 10 were reversely coded. The total score ranged from 0 to 30, with higher values indicating greater self-esteem. Scores between 15 and 25 were considered normal, whilst those below 15 suggested a lack of self-esteem.

Body size satisfaction

Perception of body size among the participants was assessed using the Nine-figure Contour Drawing Rating Scale [14]. Participants were asked to select the figure that most correctly illustrated their current and ideal body size. The difference between the current and ideal size was computed as a discrepancy score (current - ideal). A positive discrepancy score implied that the participants desired a smaller body size, while a discrepancy score of “zero” suggested that the participants were satisfied with their present body size and they were classified as “wish to maintain body size.” Participants who had a negative discrepancy score suggested that they wished for a larger body size.

Perception of bodyweight status

Participants were questioned about their perceived bodyweight status with options of significantly underweight, underweight, appropriate bodyweight, overweight or obese. Following that, the actual and perceived bodyweight statuses were compared. Participants were categorized as perceived normal weight, perceived underweight and perceived overweight.

Depression, anxiety, and stress

The Depression, Anxiety and Stress Scale (DASS-21) was used to assess emotional distress in three sub-categories: depression, anxiety and stress [15]. DASS-21 is a 21-item self-reporting questionnaire (seven for each category) with a 4-point rating scale. Each item had four answers - “did not apply to me at all,” “applied to me to some degree or some of the time,” “applied to me to a considerable degree or a good part of the time,” and “applied to me very much, or most of the time.” Each of the seven items was scored from zero to three. Total scores from each scale were multiplied by two and recommended cut-off scores for conventional severity labels were used [16]. Scoring with moderate level and above was used to determine the prevalence of depression, anxiety and stress.

Nutrition knowledge

The Nutrition Knowledge Test, developed by Sapp and Jensen, was used to measure nutrition knowledge [17]. The test consisted of 23 items designed to measure students’ knowledge of the amount of fiber (six items), energy (two items), cholesterol (four items) and fat (eight items) in various foods. Each item was dichotomously scored and featured three to five response options, including a correct answer (a score of one), incorrect answer(s) (a score of zero), and “do not know” (a score of zero). A higher score indicated a greater nutritional knowledge. KR-20 reliability coefficients for the scale were performed, which equaled to 0.69, 0.61, and 0.58, respectively; and found convergent and discriminant validity of the scale [17].

Anthropometric measurements

Bodyweight and height of the participants were carried out by trained personnel and measured using Tanita Digital Weighing Scale HD-314 (Tanita Corporation, USA) to the nearest 0.1 kg, and portable SECA stadiometer Model 213 (SECA, Germany) to the nearest 0.1 cm, respectively. Two measurements were taken for each participant to get an average value. BMI-for-age z-scores (BAZ) were computed. Bodyweight status was determined according to the WHO Growth Reference for 5-19 years [18].

Data collection

Briefing sessions were conducted to clarify the study’s objective. Participants who were interested in participating in the study were given information sheets and consent forms at the briefing session. The consent forms had to be filled out by both the participants and their parents. Upon receiving their permission to take part in the study, the researchers administered the questionnaires to the participants and assisted them when necessary. Bodyweight and height were measured during the given time. Souvenirs were given to the participants as gratitude for their valuable time. Participants who were absent were considered as non-respondents. Those who had no interest in taking part in this study and where permission was not obtained were also considered as non-respondents.

Data analysis

Data were entered into and analyzed with the Statistical Package for Social Sciences Software (SPSS) version 25.0 (SPSS Inc., Chicago, IL). Normality tests were performed prior to analyzing the data for descriptive and inferential statistics. Continuous variables were reported as mean and standard deviations, while counts and percentages were used to describe categorical variables. For group comparisons, the chi-square test and independent t-test were performed to determine the association. For the purpose of analysis, overweight and obesity were categorized into one variable “overweight or obesity” and other weight categories as “non-overweight/obesity.” The proportion of participants who were overweight or obese was computed as BAZ > +1 SD. The confidence level was set at 95% and level of significance was set at a p-value of < 0.05. Logistic regression was used to test the associations between predictors and the outcome variable. Simple logistic regression was applied in determining the crude odds ratios and variables with p < 0.25 were inserted into the multivariate logistic regression model. The predictors of obesity were determined by using the multivariate logistic regression model. The significant predictors were based on p < 0.05.

Ethical considerations

Ethical approvals were granted from Medical Research and Ethics Committee, Ministry of Health (NMRR-19-159-45671) and Ethics Committee for Research involving Human Subject, Universiti Putra Malaysia (JKEUPM-2019-264). In addition, permissions were also obtained from the Ministry of Education, District Education Office and the selected schools. Prior to data collection, the participants and their parents signed written informed consents.

## Results

Overall, 2,221 students out of the 2,659 eligible ones participated in this study, providing a total response rate of 83.5%. The rest were either not interested to participate (1.5%), had no parental consent (3.3%), or were absent during data collection (11.6%).

Characteristics of the study population

The socio-demographic, behavioral, and psychosocial characteristics, as well as the bodyweight status of the participants, are illustrated in Table 1. The mean age of adolescents who participated in the study was 14.19 years (standard deviation [SD] = 1.28) and more than half of them were female (54.5%). The majority of the participants were Malay (83.0%).

**Table 1:** Characteristics of the participating adolescents in Seremban (n=2,221)

	Male (n = 1011) n (%)	Female (n = 1210) n (%)	Overall (n = 2221) n (%)
Age groups			
< 13	226 (22.4)	255 (21.1)	481 (21.7)
13 to < 14	357 (35.3)	387 (32.0)	744 (33.5)
14 to < 15	114 (11.3)	114 (9.4)	228 (10.3)
15 to < 16	209 (20.7)	319 (26.4)	528 (23.8)
16 to < 17	105 (10.4)	135 (11.2)	240 (10.8)
Ethnicity			
Malay	852 (84.3)	992 (82.0)	1844 (83.0)
Chinese	39 (3.9)	41 (3.4)	80 (3.6)
Indian	111 (11.0)	167 (13.8)	278 (12.5)
Bumiputera Sabah	4 (0.4)	3 (0.2)	7 (0.3)
Bumiputera Sarawak	2 (0.2)	4 (0.3)	6 (0.3)
Others	3 (0.3)	3 (0.2)	6 (0.3)
Grade			
Form 1	342 (33.8)	376 (31.1)	718 (32.3)
Form 2	360 (35.6)	383 (31.7)	743 (33.5)
Form 4	309 (30.6)	451 (37.3)	760 (34.2)
Breakfast consumption			
Never	158 (15.6)	233 (19.3)	391 (17.6)
1-6 days	714 (70.6)	843 (69.7)	1557 (70.1)
Daily	139 (13.7)	134 (11.1)	273 (12.3)
Fast food consumption			
Never	106 (10.5)	127 (10.5)	233 (10.5)
1-3 days	807 (79.8)	939 (77.6)	1746 (78.6)
4-6 days	85 (8.4)	122 (10.1)	207 (9.3)
Daily	13 (1.3)	22 (1.8)	35 (1.6)
Eating out frequency			
Never	120 (11.9)	164 (13.6)	284 (12.8)
1-3 times	728 (72.0)	892 (73.7)	1620 (72.9)
4-6 times	138 (13.6)	134 (11.1)	272 (12.2)
7 times	25 (2.5)	20 (1.7)	45 (2.0)
Snacking frequency			
Never	41 (4.1)	31 (2.6)	72 (3.2)
1-3 times	672 (66.5)	802 (66.3)	1474 (66.4)
4-6 times	257 (25.4)	309 (25.5)	566 (25.5)
7 times	41 (4.1)	68 (5.6)	109 (4.9)
PAQ-C Score			
Low	487 (48.2)	788 (65.1)	1275 (57.4)
Moderate & high	524 (51.8)	422 (34.9)	946 (42.6)
Screen time (weekend)			
< 2 hours	387 (38.3)	525 (43.4)	912 (41.1)
≥ 2 hours	624 (61.7)	685 (56.6)	1309 (58.9)
Screen time (school day)			
< 2 hours	621 (61.4)	799 (66.0)	1420 (63.9)
≥ 2 hours	390 (38.6)	411 (34.0)	801 (36.1)
Healthy eating and weight self-efficacy score			
Mean ± SD	36.56 ± 6.99	37.16 ± 6.33	36.89 ± 6.65
Perceived physical activity self-efficacy score			
Mean ± SD	27.20 ± 5.08	26.87 ± 4.38	27.02 ± 4.72
Self-esteem level			
Low (<15)	179 (17.7)	206 (17.0)	385 (17.3)
Normal (≥ 15)	832 (82.3%)	1004 (83.0)	1836 (82.7)
Body discrepancy score			
Mean ± SD	0.18 ± 1.20	0.71 ± 1.35	0.46 ± 1.31
Body satisfaction			
Wish to have smaller size (discrepancy score > 0)	309 (30.6)	603 (49.8)	912 (41.1)
Wish to maintain body size (discrepancy score = 0)	481 (47.6)	440 (36.4)	921 (41.5)
Wish to have bigger size (discrepancy score < 0)	221 (21.9)	167 (13.8)	388 (17.5)
Bodyweight perception			
Perceived underweight	301 (29.8)	232 (19.2)	533 (24.0)
Perceived normal weight	440 (43.5)	504 (41.7)	944 (42.5)
Perceived overweight	270 (26.7)	474 (39.2)	744 (33.5)
Depression level			
Normal & mild (≤ 13)	609 (60.2)	767 (63.4)	1376 (62.0)
≥ Moderate (> 13)	402 (39.8)	443 (36.6)	845 (38.0)
Anxiety level			
Normal & mild (≤ 9)	369 (36.5)	418 (34.5)	787 (35.4)
≥ Moderate (> 9)	642 (63.5)	792 (65.5)	1434 (64.6)
Stress level			
Normal & mild (≤ 18)	718 (71.0)	881 (72.8)	1599 (72.0)
≥ Moderate (> 18)	293 (29.0)	329 (27.2)	622 (28.0)
Nutrition knowledge score			
Mean ± SD	10.42 ± 4.19	11.61 ± 3.64	11.07 ± 3.95
Bodyweight status			
Severe thinness	24 (2.4)	11 (0.9)	35 (1.6)
Thinness	67 (6.6)	42 (3.5)	109 (4.9)
Normal	588 (58.2)	781 (64.5)	1369 (61.6)
Overweight	162 (16.0)	215 (17.8)	377 (17.0)
Obese	170 (16.8)	161 (13.3)	331 (14.9)

Overall, the mean frequency of breakfast, fast food, eating out and snacking consumption were 2.93 (SD = 2.32), 1.92 (SD = 1.38), 2.05 (SD = 1.52), and 2.98 (SD = 1.70), respectively. The mean score of physical activity level among participants was 2.29 (SD = 0.58), with males having a higher physical activity level (2.42, SD = 0.62) than females (2.18, SD = 0.53). Females had a significantly higher total mean score of HEWSE than males, p = 0.038. On the other hand, there was no significant difference in the total mean of perceived physical activity self-efficacy between males and females. The total mean score of the self-esteem scale was 17.90 (SD = 3.71). There was no significant difference in the total mean score of self-esteem between males and females. About one-third of the males and half of the females wished to have a smaller size. Boys had a higher perception of being underweight compared to girl. In contrast, girls had a higher perception of being overweight and obese compared to boys. The total mean score of the depression, anxiety, and stress scale was 11.65 (SD = 8.78), 12.67 (SD = 8.03), and 14.75 (SD = 7.83), respectively. There was no significant difference between males and females in terms of depression, anxiety, and stress levels. Females scored a significantly higher total mean nutrition knowledge score than males, p < 0.001.

The mean body weight and height of the participants were 51.72 kg (SD = 15.39) and 155.64 cm (SD 8.40), respectively. The mean BMI was 21.17 kg/m2 (SD = 5.33) and the mean z-scores of BMI-for-age was 0.2156 (SD = 1.56). The prevalence of underweight was 6.5% and almost one-third (31.9%) of the participants were found to be either overweight or obese.

Factors associated with overweight and obesity

The association between overweight and obesity affecting factors is presented in Table 2. For behavioral factors, the frequency of breakfast, fast food, and eating out consumption, as well as physical activity level revealed a significant association with overweight and obesity. With regards to the psychosocial factors, healthy eating and weight self-efficacy, perceived physical activity self-efficacy, body satisfaction, and body size perception were significantly associated with overweight and obesity. On the other hand, the association between gender, age groups, ethnicity, snacking frequency, sedentary behavior during weekends and weekdays, self-esteem, depression, anxiety, stress, and nutrition knowledge with overweight and obesity were not found to be significant.

**Table 2:** Factors associated with overweight and obesity among the participants (n=2,221) *significant at p < 0.05; **significant at p < 0.001

Variable	Overweight & obese n (%)	Non-overweight & obese n (%)	t/Χ^2^	df	p
Sex					
Male	332 (32.8)	679 (67.2)	0.790	1	0.374
Female	376 (31.1)	834 (68.9)			
Age (years)					
< 13	155 (32.2)	326 (67.8)	8.284	4	0.082
13 to < 14	259 (34.8)	485 (65.2)			
14 to < 15	75 (32.9)	153 (67.1)			
15 to < 16	144 (27.3)	384 (72.7)			
16 to < 17	75 (31.3)	165 (68.8)			
Ethnicity					
Malay	604 (32.8)	1240 (67.2)	6.840	5	0.233
Chinese	20 (25.0)	60 (75.0)			
Indian	81 (29.1)	197 (70.9)			
Bumiputera Sabah	2 (28.6)	5 (71.4)			
Bumiputera Sarawak	0 (0.0)	6 (100.0)			
Others	1 (16.7)	5 (83.3)			
Breakfast consumption					
Never	152 (38.9)	239 (61.1)	23.035	2	<0.001**
1-6 days	498 (32.0)	1059 (68.0)			
Daily	58 (21.2)	215 (78.8)			
Fast food consumption					
Never	93 (39.9)	140 (60.1)	10.889	3	0.012*
1-3 days	550 (31.5)	1196 (68.5)			
4-6 days	53 (25.6)	154 (74.4)			
Daily	12 (34.3)	23 (65.7)			
Eating out frequency					
Never	107 (37.7)	177 (62.3)	11.260	3	0.010*
1-3 times	518 (32.0)	1102 (68.0)			
4-6 times	67 (24.6)	205 (75.4)			
7 times	16 (35.6)	29 (64.4)			
Snacking frequency					
Never	25 (34.7)	47 (65.3)	4.876	3	0.181
1-3 times	432 (30.3)	996 (69.7)			
4-6 times	214 (34.7)	402 (65.3)			
7 times	37 (35.2)	68 (64.8)			
Physical activity					
Low	458 (35.9)	817 (64.1)	22.545	1	<0.001**
Moderate & high	250 (26.4)	696 (73.6)			
Screen time (weekend)					
< 2 hours	279 (30.6)	633 (69.4)	1.177	1	0.278
≥ 2 hours	429 (32.8)	880 (67.2)			
Screen time (school day)					
< 2 hours	458 (32.3)	962 (67.7)	0.256	1	0.613
≥ 2 hours	250 (31.2)	551 (68.8)			
Self-esteem					
Low (< 15)	120 (31.2)	265 (68.8)	0.108	1	0.743
Normal (≥ 15)	588 (32.0)	1248 (68.0)			
Body size satisfaction					
Maintain	202 (21.9)	719 (78.1)	412.894	2	<0.001**
Wish to have smaller size	497 (54.5)	415 (45.5)			
Wish to have bigger size	9 (2.3)	379 (97.7)			
Body size perception					
Perceived underweight	75 (14.1)	458 (85.9)	501.933	2	<0.001**
Perceived normal weight	164 (17.4)	780 (82.6)			
Perceived overweight	469 (2.3)	275 (37.0)			
Depression					
Normal & mild (≤ 13)	422 (30.7)	954 (69.3)	2.434	1	0.119
≥ Moderate (> 13)	286 (33.8)	559 (66.2)			
Anxiety					
Normal & mild (≤ 9)	253 (32.1)	534 (67.9)	0.041	1	0.840
≥ Moderate (> 9)	455 (31.7)	979 (68.3)			
Stress					
Normal & mild (≤ 18)	511 (32.0)	1088 (68.0)	0.017	1	0.897
≥ Moderate (> 18)	197 (31.7)	425 (68.3)			
Healthy eating and weight self-efficacy					
Mean ± SD	36.34 ± 6.42	37.14 ± 6.74	-2.634	2219	0.009*
Perceived physical activity self-efficacy					
Mean ± SD	26.72 ± 4.73	27.16 ± 4.70	-2.075	2219	0.038*
Nutrition knowledge					
Mean ± SD	11.23 ± 4.11	11.00 ± 3.87	1.249	1308.78	0.202

Predictors of overweight and obesity

Table 3 shows the logistic regression analysis of selected factors with overweight and obesity among participants. The probability of being overweight and obese was estimated using variables that had p < 0.25 in the simple logistic regression. Breakfast consumption, physical activity level, healthy eating, weight self-efficacy, perceived physical activity self-efficacy, body dissatisfaction, and body size perception were retained for multivariate logistic regression analysis. Breakfast skippers were 1.73 times more likely of being overweight and obese than those who ate breakfast, AOR = 1.73, 95% CI [1.141, 2.628], p = 0.010. Participants with low levels of physical activity were 1.37 times more likely of being overweight and obese, AOR = 1.37, 95% CI [1.095, 1.701], p = 0.006, than those who have moderate and high physical activity levels. Those who wished to have a smaller body size were 2.56 times more likely of being overweight and obese, AOR = 2.56, 95% CI [2.035, 3.217], p < 0.001, than those who were satisfied with their body. On the other hand, those who wished to have a bigger body size were less likely to be overweight and obese, AOR = 0.11, 95% CI [0.055, 0.218], p < 0.001, than those who were satisfied with their body. The analysis revealed that the perception of large body size has significantly increased the odds of being overweight and obese by 4.40 times, AOR = 4.40, 95% CI [3.184, 6.080], p < 0.001, compared to those who perceived themselves as underweight. There was a negative association between self-efficacy and being overweight and obese. For each unit increase in healthy eating and weight self-efficacy score, the likelihood of being overweight and obese decreases, AOR = 0.98, 95% CI [0.966, 0.999], p = 0.045. Likewise, for each unit increase in perceived physical activity self-efficacy score, the probability of being overweight and obese also decreases, AOR = 0.98, 95% CI [0.952, 0.999], p = 0.039.

**Table 3:** Logistics regression analysis on predictors of overweight and obesity *significant at p < 0.05; **significant at p < 0.001

Variables	Simple logistic regression			Multivariate logistic regression		
	Crude OR	95% CI	p	AOR	95% CI	p
Breakfast consumption						
0	2.358	[1.654, 3.360]	<0.001**	1.731	[1.141, 2.628]	0.010*
1-6 days	1.743	[1.280, 2.374]	<0.001**	1.528	[1.064, 2.194]	0.022*
7 days	1			1		
Fast food consumption						
0	1.273	[0.604, 2.684]	0.525			
1-3 days	0.881	[0.435, 1.784]	0.726			
4-6 days	0.660	[0.307, 1.417]	0.286			
7 days	1					
Eating out						
0	1.096	[0.569, 2.111]	0.785			
1-3 days	0.852	[0.459, 1.583]	0.612			
4-6 days	0.592	[0.303, 1.157]	0.125			
7 days	1					
Physical activity level						
Low	1.561	[1.298, 1.877]	<0.001**	1.365	[1.095, 1.701]	0.006*
Moderate & high	1			1		
Healthy eating and weight self-efficacy	0.982	[0.969, 0.995]	0.009*	0.983	[0.966, 0.999]	0.045*
Perceived physical activity self-efficacy	0.980	[0.962, 0.999]	0.038*	0.975	[0.952, 0.999]	0.039*
Body satisfaction						
Wish to have smaller size	4.263	[3.478, 5.224]	<0.001**	2.559	[2.035, 3.217]	<0.001**
Wish to have bigger size	0.085	[0.043, 0.167]	<0.001**	0.109	[0.055, 0.218]	<0.001**
Satisfied	1			1		
Body size perception						
Perceived overweight	10.415	[7.825, 13.862]	<0.001**	4.399	[3.184, 6.080]	<0.001**
Perceived normal weight	1.284	[0.954, 1.727]	0.099	0.836	[0.606, 1.154]	0.276
Perceived underweight	1			1		

## Discussion

About 17.0% of the participants in this study were overweight and another 14.9% were obese. The prevalence of overweight and obesity found in this study was slightly higher compared to the National Health and Morbidity Survey in 2017 (15.6% and 14.8%, respectively) and 2019 (15.0% and 14.8%, respectively) [7,9]. In this study, the prevalence of overweight and obesity was higher than the pooled prevalence in low-income and middle-income countries and Asian countries [19,20]. The burden of a high prevalence of excess weight among adolescents emphasizes the need to broaden the scope of nutrition guidelines as well as public health policies and programs to address overweight and obesity in Malaysia.

Employing the multivariate model, breakfast consumption, physical activity level, healthy eating, weight self-efficacy, perceived physical activity self-efficacy, body dissatisfaction, and body perception remained significant, while the rest failed to show such significant associations. The study did not find a significant relationship between socio-demographic characteristics with overweight and obesity. The findings appear to differ from other study reported that girls had significantly higher BMI-for-age compared to boys [21], while some reported a reverse pattern [22]. The proportion of participants who reported being overweight and obese did not differ by age group and ethnic group. This could be due to the narrow age group comparison and ethnicity imbalance in this study.

The current study explored the patterns of breakfast consumption and its association with overweight and obesity. The results from this study highlighted that regular breakfast intake was associated with a lower likelihood of overweight and obesity among the participants. The finding was consistent with some local studies, for instance, the MyBreakfast Study, where the proportion of overweight and obesity was significantly higher among breakfast-skippers and irregular breakfast eaters compared to regular breakfast eaters [23]. As this was a cross-sectional study, the findings might potentially indicate that those who were overweight and obese tried to avoid eating breakfast in order to skip meals and lose weight.

In this study, consumption of fast food, eating out, and snacking were not significantly predicting the likelihood of being overweight and obese. Interestingly, a higher frequency of these three meal patterns was observed among participants who were normal weight and thin; however, the difference was not significant. This might be due to the increased awareness of childhood obesity in this country, which could have led to the under-reporting of dietary intake due to social desirability bias. In this study, food selection during eating out was not studied, therefore healthier food choices during eating out were not determined. In line with previous studies, this study reported a significant association between low physical activity and higher odds of overweight and obesity in adolescents [24,25]. On the other hand, a sedentary time during weekends and weekdays was not significantly associated with overweight and obesity in this study.

The current study demonstrated a borderline association between low self-efficacy (in terms of healthy eating and weight, and perceived physical activity) and higher odds of overweight and obesity. The finding in this study is contrary to a previous study, which revealed that there was no difference between groups in scores for autonomous motivation factors for eating a healthy diet, autonomous motivation factors for exercise, controlled motivation for exercise, and perceived competence in exercising [26]. Mixed results were also reported, where the overweight and the obese group scored significantly lower in physical activity self-efficacy but no significant difference in eating self-efficacy as compared to the normal weight group [24].

Body satisfaction and body size perception were significantly associated with overweight and obesity in this study. Overweight and obese participants were reported to have significantly higher body discrepancy scores as compared with their counterparts. About 71.5% of the overweight and obese participants were dissatisfied with their body as compared with 52.5% of participants who had normal weight and were thin. This finding was consistent with other studies [21,27]. In this study, about 33.8% of the participants from the overweight and obese group underestimated their body weight status, while about 18.2% of the participants from the non-overweight group overestimated their body weight status. As compared with other studies, among overweight and obese children, more than one-third was not aware that he or she was overweight [28]. In addition, it was reported that among underweight children, 72.0% of them perceived themselves at a higher body size, while 83.0% of overweight and obese children perceived themselves at a lower body size [27].

In this study, results revealed that the proportion of participants who reported being overweight and obese did not significantly differ by self-esteem, depression, anxiety, stress, and nutrition knowledge. This finding was inconsistent with previous studies, where self-esteem scores of the overweight and obese groups were significantly lower than those of the normal weight students [21], and students who felt sad, hopeless, lonely most of the time, could not stop worrying and had sleeplessness most of the time were statistically more likely to be overweight [29]. On the other hand, several other studies showed that there was no relationship between the complexity of knowledge about healthy eating and BMI [24,30].

The strength of this study is that the sample size was adequate. Besides, objective measurements of body weight and height were conducted by trained persons as opposed to self-reported anthropometric data. However, the study has several limitations. For instance, due to the study design being cross-sectional, no causal implications could be drawn. Adolescents’ self-reported data could present its own set of problems, including social desirability, recall bias, and misreporting. In addition, there was no use of food frequency questionnaires, 24-hour food recall, or food records in this study. Data on important confounders (for example parental factors, health-risk behavior such as smoking habits, alcohol consumption) were not collected. All environmental settings and factors that potentially impact childhood obesity, such as the quantity and quality of physical education at the school level and home, the school nutrition program as well as school resources were also not investigated. Moreover, the influence of contextual variables at the community level, such as availability and accessibility of a safe park or playground, accessibility to healthy food in the community were not investigated.

## Conclusions

The study was aimed at determining the prevalence and predictors of overweight and obesity among adolescents in Seremban, Negeri Sembilan, Malaysia. It was found that 17.0% and 14.9% of adolescents were overweight and obese, respectively. Overweight and obesity are emerging public health issues among Malaysian school children. The significant predictors of overweight and obesity identified in this study included breakfast skipping, low physical activity level, low healthy eating and weight self-efficacy scores, low perceived physical activity self-efficacy scores, body dissatisfaction and body perception. The findings in this study highlighted the need of healthy eating habits, such as eating breakfast in overweight and obesity prevention programs. Such program may include interventions to increase physical activity and reduce sedentary behavior among youth, such as providing enhanced physical education class, classroom activity breaks, and after-school activities, as well as encouraging active transportation to school. The finding also suggests that overweight and obesity prevention program in adolescents need to focus on self-efficacy, especially in increasing confidence to carry out healthy eating habits and weight control, as well as performing exercise or physical activity. In addition, health education programs need to include topics on body image, body satisfaction and methods of weight control. Although depression and anxiety did not significantly associate with body weight status, it is still crucial to be addressed among adolescents in promoting psychological well-being. These findings could highlight the need for coping strategies in interventions to tackle depression and anxiety at its very early stages.
